# An application of a pattern-mixture model with multiple imputation for the analysis of longitudinal trials with protocol deviations

**DOI:** 10.1186/s12874-018-0639-y

**Published:** 2019-01-09

**Authors:** Abdul-Karim Iddrisu, Freedom Gumedze

**Affiliations:** Department of Statistical Sciences, University of Cape Town, Cape Town, Rondebosch7701 South Africa

**Keywords:** Likelihood-based methods, Missing at random, Multiple imputation, Not missing at random, Pattern-mixture model, Protocol deviation, Sensitivity analysis

## Abstract

**Background:**

The benefit of a given treatment can be evaluated via a randomized clinical trial design. However, protocol deviations may severely compromise treatment effect since such deviations often lead to missing values. The assumption that methods of analysis can account for the missing data cannot be justified and hence methods of analysis based on plausible assumptions should be used. An alternative analysis to the simple imputation methods requires unverifiable assumptions about the missing data. Therefore sensitivity analysis should be performed to investigate the robustness of statistical inferences to alternative assumptions about the missing data.

**Aims:**

In this paper, we investigate the effect of tuberculosis pericarditis treatment (prednisolone) on CD4 count changes over time and draw inferences in the presence of missing data. The data come from a multicentre clinical trial (the IMPI trial).

**Methods:**

We investigate the effect of prednisolone on CD4 count changes by adjusting for baseline and time-dependent covariates in the fitted model. To draw inferences in the presence of missing data, we investigate sensitivity of statistical inferences to missing data assumptions using the pattern-mixture model with multiple imputation (PM-MI) approach. We also performed simulation experiment to evaluate the performance of the imputation approaches.

**Results:**

Our results showed that the prednisolone treatment has no significant effect on CD4 count changes over time and that the prednisolone treatment does not interact with time and anti-retroviral therapy (ART). Also, patients’ CD4 count levels significantly increase over the study period and patients on ART treatment have higher CD4 count levels compared with those not on ART. The results also showed that older patients had lower CD4 count levels compared with younger patients, and parameter estimates under the MAR assumption are robust to NMAR assumptions.

**Conclusions:**

Since the parameter estimates under the MAR analysis are robust to NMAR analyses, the process that generated the missing data in the CD4 count measurements is missing at random (MAR). The implication is that valid inferences can be obtained using either the likelihood-based methods or multiple imputation approaches.

## Background

The benefit of trial medication may be evaluated through a randomized clinical trials design. Randomized clinical trials with longitudinal follow-up are central to the evaluation of treatments. However, statistical inferences from the resulting analysis is almost always complicated because subjects might deviate from the protocol [1]. The study protocol sets out the objective and procedure of conducting the trial.

Given the trial setting and the specific question, such deviations may include poor compliance with, or withdrawal from the intervention; unblinding, either of intervention or evaluation; and loss to follow-up, so that no further information on the patient is available [1]. These deviations complicate the analysis because (to address both primary and secondary questions), there is the need to make assumptions about the unobserved data [1, 2]. These assumptions are often not verifiable. There is now an increase in awareness that such assumptions have the potential to introduce implicit ambiguity into the inferences that can be drawn [1, 3, 4]. In addition, inappropriate assumptions about the unobserved data may lead to biased estimates of the treatment effect. The extent to which such inappropriate assumptions are practical issues will depend both on the precise question, and on how the extent and nature of deviations from the protocol affect this question [1]. Most often, regulators and analysts will require some level of confidence that inferences are robust to plausible departures from the primary assumptions that govern the main analysis. This gives an indication that such inferences require sensitivity analyses [1].

It is known that missing data may severely compromise statistical inferences from clinical trials. However, missing data has received little attention in the clinical-trials research [5] and existing regulatory guidelines [6] on design, conduct, and analysis of clinical trials have limited advice on how to handle missing data. The national research council (NRC) report [3] outlined recommendations for handling missing data in clinical trials.

There is now increasing attention to the importance of conducting sensitivity analysis in the biomedical research. For instance, Section 7 of the new EMA guideline on missing data in confirmatory clinical trials [7] is devoted to this issue. It states “The sensitivity analyses should show how different assumptions influence the results obtained.” In addition, recommendation 15 of the NRC report [3] recommended that “sensitivity analyses should be part of the primary reporting of findings from clinical trials. The sensitivity to the assumptions about the missing data mechanism should be a mandatory component of reporting.”

Sensitivity to missing data can be conducted based on three modeling frameworks [8]. In the selection modeling (SeM) framework, the joint distribution of the measurement and the dropout processes is factored as the marginal distribution of the measurement process and the conditional distribution of the dropout process, given the measurements [8, 9]. The pattern-mixture model (PMM) is a reverse factorization of the SeM defined as the marginal distribution of the dropout process and the conditional distribution of the measurement process given the dropout process [2, 8]. For the shared-parameter model (SPM), a set of latent variables (random effects) is assumed to be shared between the measurement and the dropout processes [8, 10]. It is conventionally assumed that conditional on this set of random effects, no further dependency exists between the measurement and the dropout process, although this can be generalized [11]. Yuan and Little [12] proposed mixed-effect hybrid models (MEHMs) framework, where the joint distribution of the measurement process and dropout process is factorized into the marginal distribution of random effects, the dropout process conditional on random effects, and the outcome process conditional on dropout patterns and random effects. Carpenter and colleagues [1] proposed the pattern-mixture model with multiple imputation (PM-MI) approach in order to conduct sensitivity analysis. Ratitch and colleagues [13] considered sensitivity analysis approaches based on the pattern-mixture model, and Mallinckrodt and colleagues [14] considered selection model based approaches [9] and the PM-MI approach [1] to conduct sensitivity analyses. Permutt and colleagues [15] examined previous ideas of sensitivity analysis with a view to explaining how the NRC panel’s recommendations are different and possibly better suited to coping with present problems of missing data in the regulatory setting. They also discussed, in more detail than the NRC report, the relevance of sensitivity analysis to decision-making, both for researchers and for regulators. In this paper, we applied the PM-MI approach of Carpenter and colleagues to investigate the effect of prednisolone treatment on CD4 count changes over time and to investigate sensitivity of inferences to missing data assumptions [8, 16, 17] using the incomplete CD4 count data from the IMPI trial [18, 19]. Carpenter and colleagues [1] applied the PM-MI approach to longitudinal data but used models which assumed independent observations, i.e., they fitted models to values at the last visit, whereas in the models in this paper we considered CD4 count measurements at all visits.

In “Description of the IMPI trial data” section, we give a brief description of the IMPI trial data. In “Estimands for primary and sensitivity analyses” section, we define the estimands and their associated deviations as well as some key assumptions relevant for the PM-MI approach. In “Standard pattern-mixture model and the pattern-mixture model with multiple imputation” section, we briefly review the standard pattern-mixture model and then discuss the pattern-mixture model with multiple imputation (PM-MI) [1]. This is followed by a brief discussion of the assumptions (sensitivity analysis) that allow us to obtain missing post-deviation data under the PM-MI approach in “Constructing joint distributions of pre-deviation and post-deviation outcome data” section. We then applied the PM-MI approach to the incomplete CD4 count data from the IMPI trial in “Application of the PM-MI approach to the IMPI trial CD4 count data” section. In “Simulation study” section, we perform simulation studies to evaluate the performance of the PM-MI approach. Finally, we give a discussion of the results and concluding remarks in “Discussion and conclusion” section.

## Description of the IMPI trial data

In this paper, we used data from the IMPI trial [18, 19]. The IMPI trial was a multicentre international randomized doubled-blind placebo-controlled 2 × 2 factorial study. The IMPI trial tested prednisolone and Mycobacterium indicus pranii (M. indicus pranii) immunotherapy treatments in TB pericarditis patients in Africa. TB pericarditis leads to high mortality especially in countries with limited resource and with concomitant epidemics of human immunodeficiency virus (HIV) infection [18, 19]. Tuberculous pericarditis is associated with high morbidity and mortality even if anti-tuberculosis treatment is taken as directed [19]. A reduction in the strength of the inflammatory response in TB pericarditis may improve patients conditions by reducing cardiac tamponade and pericardial constriction. However, whether the use of adjunctive immunomodulation with corticosteroids and M. indicus pranii can safely reduce mortality and morbidity is uncertain [19]. To investigate whether adjunctive immunomodulation with corticosteroids and M. indicus pranii can safely reduce mortality and morbidity, Mayosi and colleagues set up the IMPI trial [18, 19].

In total, 1400 patients with definite probable tuberculosis pericardial effusion, from 9 African countries in 19 centers were enrolled in the four-year trial. Eligible patients were randomly assigned to receive oral pill prednisolone for 6 weeks and M. indicus pranii or placebo for 3 months. Patients were followed up at weeks 2, 4, and 6 and months 3 and 6 during the intervention period and 6-monthly thereafter for up to 4 years [18].

The main aim of the IMPI trial was to assess the effectiveness and safety of oral pill prednisolone and M.w injection in reducing the time to first occurrence of the primary composite outcome of death, pericardial constriction, or cardiac tamponade requiring pericardial drainage in with TB pericardial effusion [19]. In this paper, we assessed the effect of trial medication (prednisolone) on CD4 count changes over time. A large proportion of the TBP patients were also co-infected with HIV (42%). Hence the interest in investigating the effect of prednisolone among HIV positive (denoted as HIV+) patients. We restricted our analysis to HIV positive (denoted as HIV+) patients only who have at least two CD4 count values observed. In the IMPI trial, patients who were confirmed HIV+ at the time of randomization or confirmed to be HIV+ during the trial, were given a standard of care (ART) and their CD4 count were measured at some visits. Mayosi and colleagues [19] results showed that the oral pill prednisolone and M. indicus pranii do not interact and hence, treatments arms were analyzed separately with their corresponding placebo arms. Also, their results showed that prednisolone reduces the risk of constriction whereas M. indicus pranii was not effective. We considered analysis of the CD4 count measurements under the prednisolone treatment and its corresponding placebo arm only. The analysis of CD4 count data is restricted to the mandated periods for CD4 count measurements; baseline, week 2, months 1, 3 and 6. However, most South Africa centres continued to measure CD4 count at months 24, 36 and 48 scheduled visit time. These data were excluded in this analysis. A majority of patients had unobserved CD4 count with 72%, 84%, 93% as missingness proportions for the months 24, 36 and 48, respectively.

In this paper, we applied the PM-MI approach to non-monotone and monotone missing data patterns. For non-monotone missing data pattern, patients can be missing at any scheduled visit and then be observed at the subsequent visit. For monotone missing data pattern, if the *i*^th^ patient is missing at schedule visit *j*, then this same patient will be missing at the next scheduled visit *j*C1.

### Non-monotone data

**Fig. 1:**
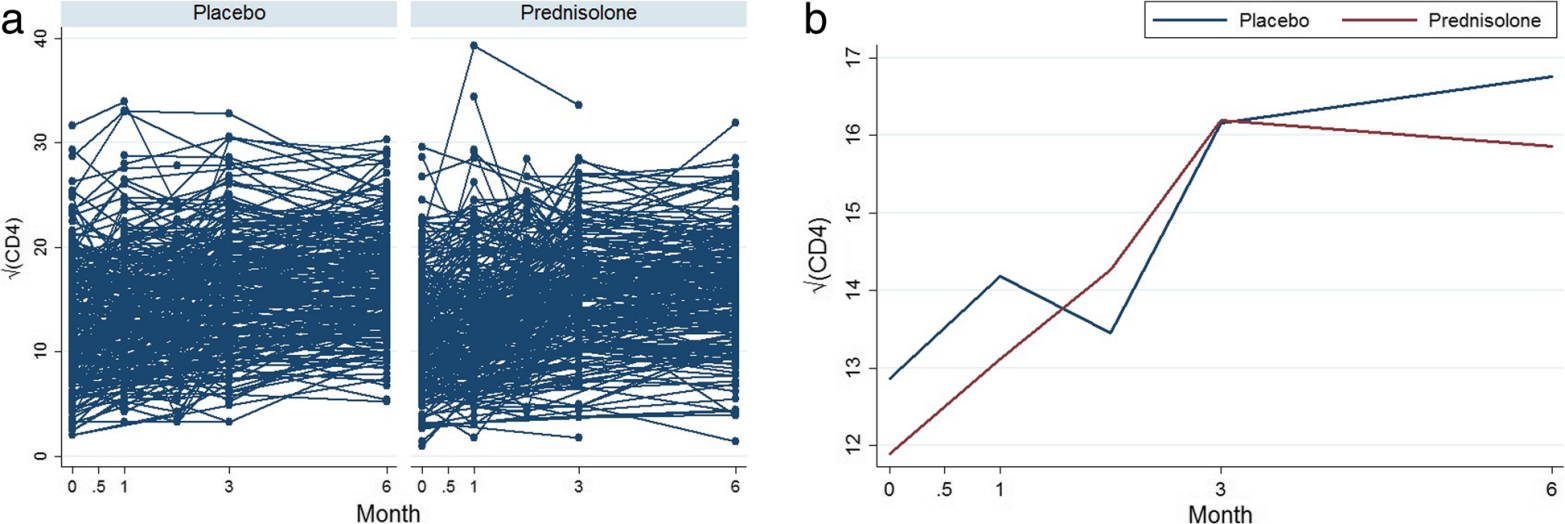
Profiles plots of the non-monotone $\sqrt {\text {CD4}}$ count data (left panel) and the mean $\sqrt {\text {CD4}}$ count (right panel), by placebo and prednisolone treatment arms

**Fig. 2:**
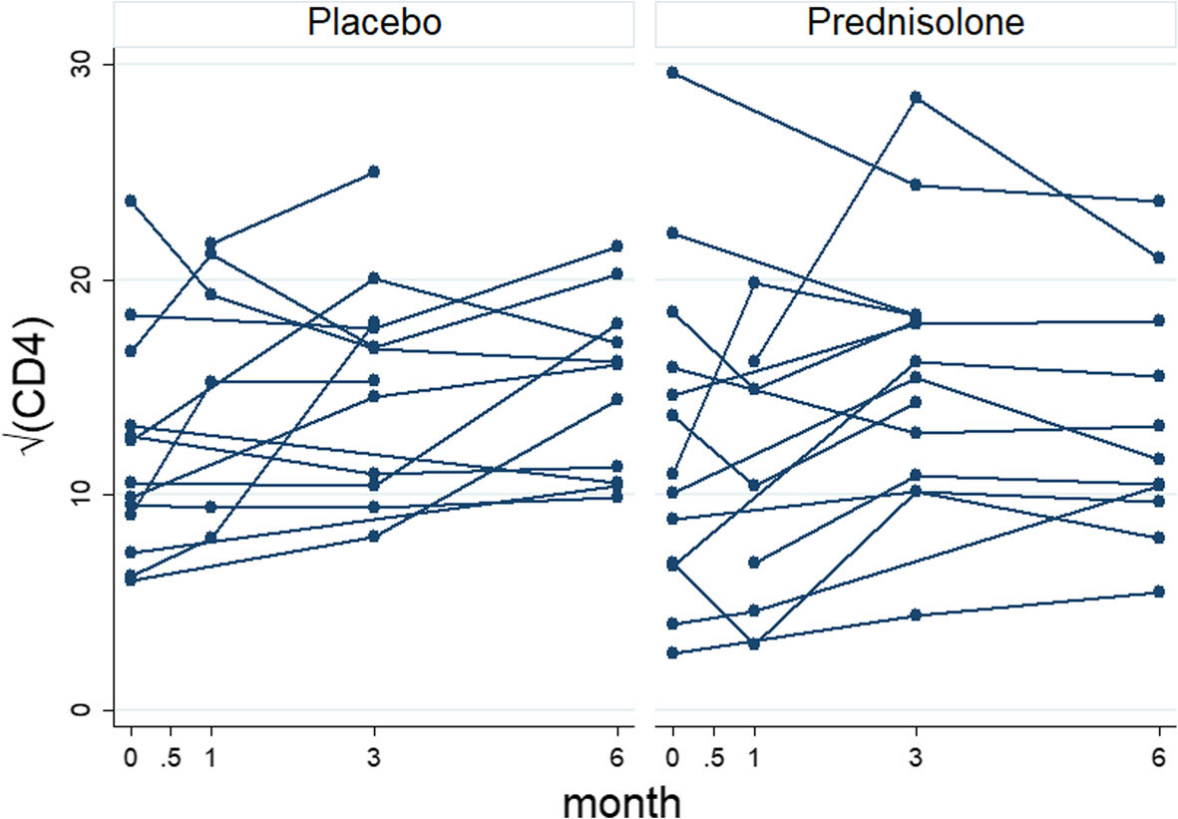
Observed profiles plots of the non-monotone $\sqrt {\text {CD4}}$ count data for 29 (5%) patients, by placebo and prednisolone treatment arms

**Fig. 3:**
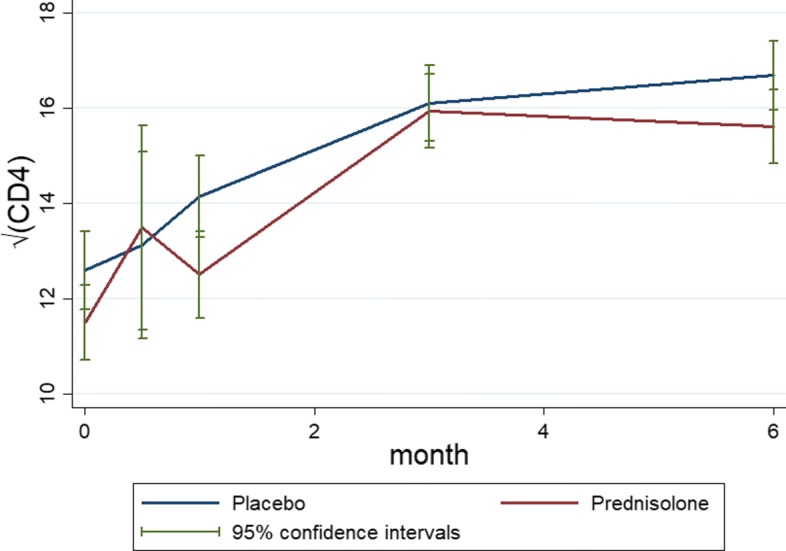
Mean profiles plots with standard error bars of the non-monotone $\sqrt {\text {CD4}}$ count data by placebo and prednisolone treatment arms

Out of 587 HIV+ patients, 294 patients are in the placebo arm and 293 patients are sin the prednisolone arm. Some of the patients have missing values within the selected scheduled visits. The left panel of Fig. 1 shows profiles plots of the observed $\sqrt {\text {CD4}}$ count measurements for each patient. Some of the patients CD4 count values are missing at either months 0.5, 1, or 3, after the baseline measurements are taken whereas some patients completed the study with their values observed from baseline up to month 6. Because there are too many patients in the left panel of the Fig. 1, the figure is not that informative. We have provided observed profiles plots of 29 (5%) patients in Fig. 2 to make this panel more informative. It can be observed from these plots that some patients completed the study (observed from baseline 0 to the month 6) while others have missing values (incomplete cases). The right panel of the Fig. 1 shows the profiles plots of the mean $\sqrt {\text {CD4}}$ count measurements by treatment arms. The mean profile plots showed a slight reduction of CD4 count level among patients in the prednisolone arm compared with those in the placebo arm. The Fig. 3 displays standard error bars around the mean graph. These plots show an overlap between confidence intervals which suggests comparable ART benefit to patients in the placebo and the prednisolone arms. There are 25 missingness patterns, presented in Table 1. A missingness pattern represents time points for which a group of patients values are missing or observed at all time points. The Table 1 shows the mean $\sqrt {\text {CD4}}$ count for each of the missingness patterns at each visit by treatment arm. The proportion of patients with missing values, in the prednisolone arm (84%), is approximately the same to that of the patients in the placebo arm (85%). Table 1 presents summaries of the $\sqrt {\text {CD4}}$ count data by treatment groups. The distribution of the pattern of missingness between the two treatment groups does not differ (chi-squared test statistic D 29.97, *p* D 0.1858).

**Table 1:** Non-monotone data: mean $\sqrt {\text {CD4}}$ count at each visit by treatment arm

	Visit (month)					
Missingness pattern ^[*a*]^	0	0.5	1	3	6	N (%)
Placebo arm						
1	13.14	13.47	13.62	16.24	17.09	44 (15)
2	12.58	13.70	14.76	17.01	-	9 (3)
3	16.90	17.68	20.27	-	-	4 (1.4)
4	16.00	12.44	-	-	-	7 (2.4)
5	11.85	13.06	13.95	-	14.12	7 (2.4)
6	12.19	-	-	-	14.87	13 (4.4)
7	12.60	13.49	-	16.92	20.60	6 (2)
8	10.65	-	-	13.09	14.90	15 (5)
9	-	-	-	15.59	17.33	4 (1.4)
10	13.17	-	14.29	16.92	17.19	90 (31)
11	-	-	16.64	16.92	17.62	16 (5.4)
12	-	13.78	13.77	15.77	16.17	12 (4.1)
13	11.15	-	12.96	-	15.85	9 (3.1)
14	10.77	10.17	-	10.67	-	3 (1)
15	12.89	-	13.20	15.65	-	18 (6)
16	10.64	-	-	13.44	-	5 (1.7)
17	13.61	-	12.46	-	-	10 (3.4)
18	-	11.33	13.65	17.70	-	4 (1.4)
19	-	13.36	-	14.98	16.76	8 (2.7)
20	-	-	-	-	-	0 (0)
21	-	-	-	-	-	0 (0)
22	-	-	13.00	16.31	-	6 (2)
23	-	-	14.64	-	15.62	3 (1)
24	-	18.19	-	14.04	-	1 (0.3)
25	-	18.03	19.52	-	15.36	1 (0.3)
All patients mean (std)	12.86 (0.361)	13.45 (0.533)	14.18 (0.368)	16.17 (0.349)	16.77 (0.334)	294 (100)
Prednisolone arm						
1	13.18	15.52	14.70	16.76	16.822	46 (16)
2	19.89	19.27	19.51	21.17	-	5 (2)
3	9.04	12.26	12.49	-	-	12 (4.1)
4	11.64	11.43	-	-	-	10 (3.4)
5	2.83	3.61	3.32	-	4.47	1 (0.3)
6	9.62	-	-	-	14.15	11 (4)
7	13.84	16.89	-	18.47	18.12	4 (1.4)
8	11.06	-	-	14.57	14.29	28 (10)
9	-	-	-	19.10	19.57	5 (2)
10	12.42	-	13.03	15.83	16.09	76 (26)
11	-	-	11.85	16.16	15.82755	13 (4.4)
12	-	12.27	13.99	15.88	15.66	14 (5)
13	9.07	-	9.26	-	15.92	12 (4.1)
14	-	-	-	-	-	0 (0)
15	12.52	-	13.21	15.54	-	14 (5)
16	14.12	-	-	15.73	-	9 (3.1)
17	8.12	-	10.74	-	-	13 (4.4)
18	-	14.35	14.73	16.00	-	2 (0.7)
19	-	17.10	-	20.95	21.59	4 (1.4)
20	-	15.07	13.10	-	-	3 (1)
21	-	10.21	-	-	10.80	2 (0.7)
22	-	-	15.07	16.78	-	3 (1)
23	-	-	13.09	-	9.32	3 (1)
24	-	18.19	-	14.04	-	1 (0.3)
25	-	8.35	8.20	-	13.36	2 (0.7)
All patients mean (std)	11.89 (0.333)	106, 14.27 (0.575)	219, 13.12 (0.397)	224, 16.20 (0.344)	221, 15.86 (0.348)	293 (100)

^a^Missingness patterns: 1 = CD4 count data at all visits, 2 = CD4 count data at all visits except 6, 3 = CD4 count data at all visits except 6 and 3, 4 = CD4 count data at all visits except 6, 3, and 1, 5 = CD4 count data at all visits except visit 3, 6 = CD4 count data at baseline and visit 6 etc

### Monotone data

The monotone CD4 count data consisted of 137 HIV positive patients. 64 were in the placebo arm and 73 in the prednisolone arm.

**Fig. 4:**
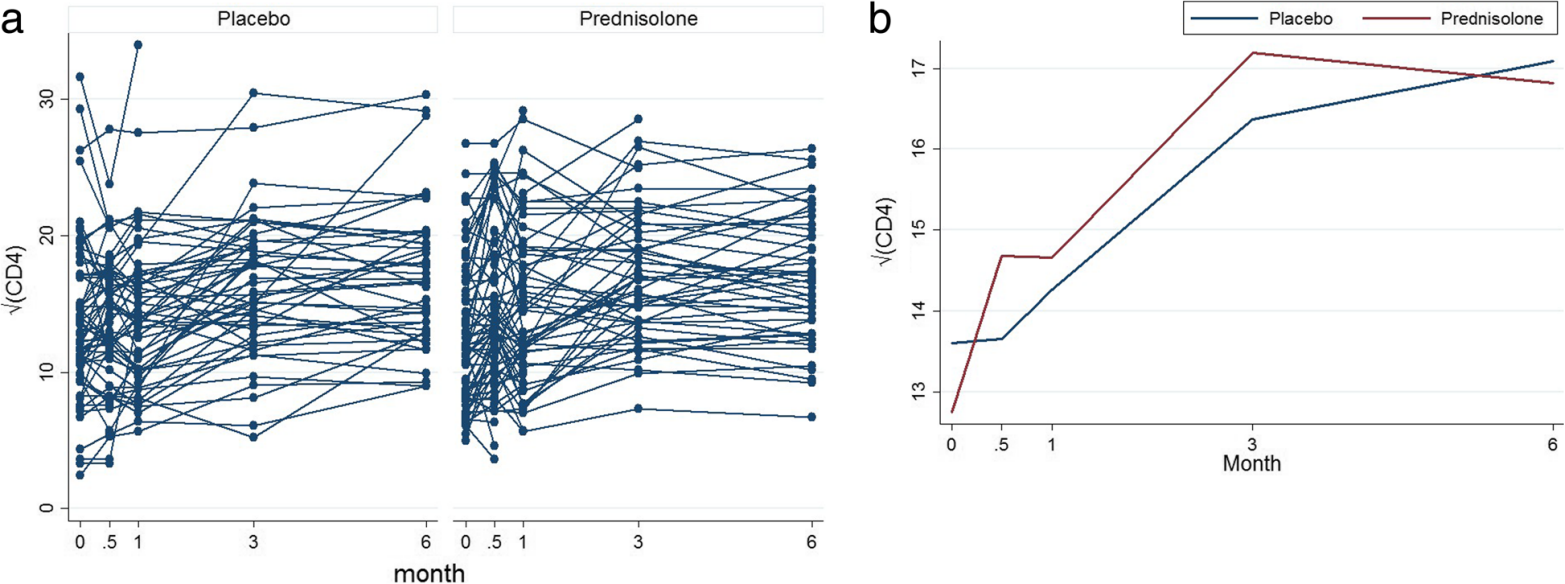
Profiles plots of the monotone $\sqrt {\text {CD4}}$ count data (left panel) and the mean $\sqrt {\text {CD4}}$ count (right panel), by placebo and prednisolone treatment arms

**Fig. 5:**
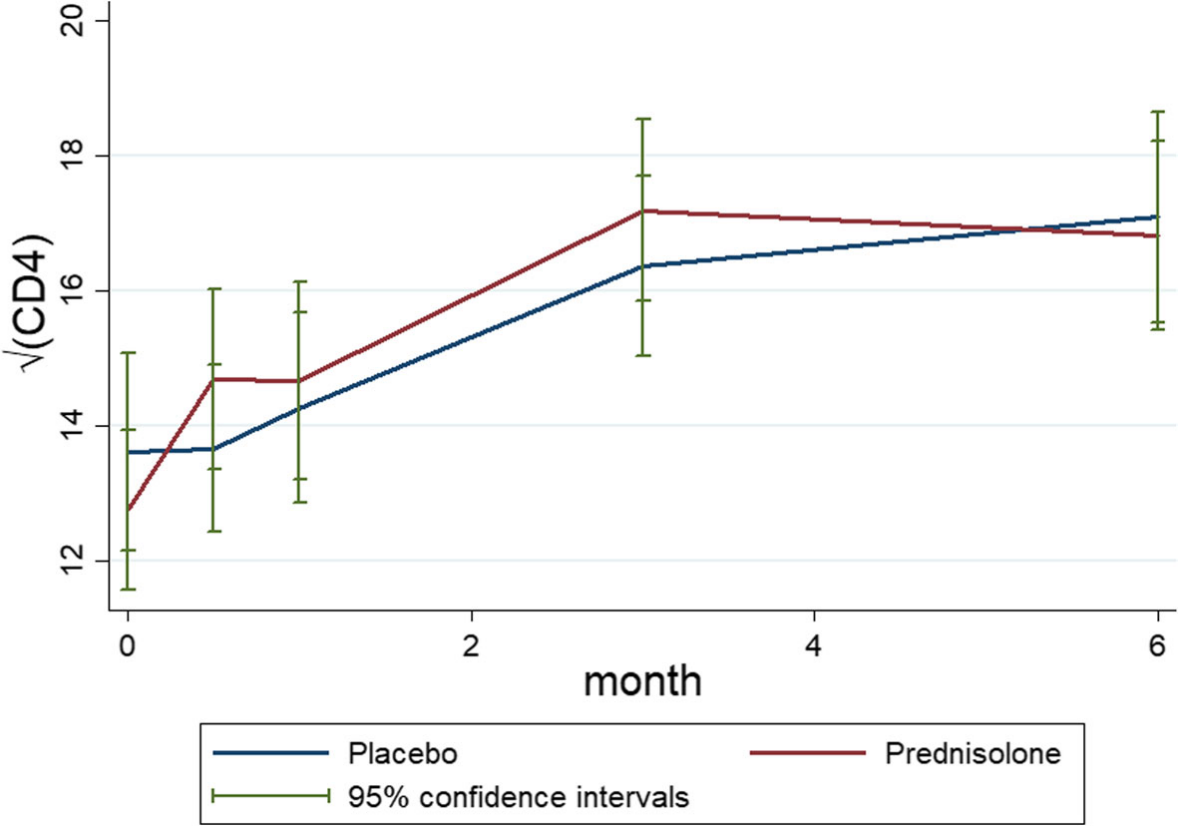
Mean profiles plots with standard error bars of the monotone $\sqrt {\text {CD4}}$ count data by placebo and prednisolone treatment arms

The left panel of Fig. 4 shows profiles plots of the observed $\sqrt {\text {CD4}}$ count measurements for each HIV positive patient. Some of these patients dropped out at months 0.5, 1, and 3, after the baseline measurements are taken, whereas some patients completed the study with their values observed from baseline up to month 6. The right panel of the Fig. 4 shows profiles plots of the mean $\sqrt {\text {CD4}}$ count measurements by treatment arms, where it can be observed that there is a slight reduction of CD4 count level for patients in the prednisolone arm compared with those in the placebo arm. The Fig. 5 displays standard error bars around the mean graph. These plots show an overlap between confidence intervals which suggests comparable ART benefit to patients in the placebo and the prednisolone arms.

Table 2 gives the number and proportion of patients remaining at each visit by treatment arm. There is higher completion rate 44 (69%) in the placebo arm compared with 46 (63%) completion rate in the prednisolone treatment arm. There are four deviation patterns. A deviation pattern represents the time point for which a group of patients dropped out of the study. The deviation patterns 4, 3, 2 and 1 represent completers (those patients who completed the study without missing values), those who dropped out at months 3, 1 and 0.5 respectively. Table 3 shows the mean $\sqrt {\text {CD4}}$ count for each of the deviation patterns at each visit by treatment arm. The proportion of patients deviating (who do not complete the study) in the prednisolone arm (37%) is higher than the portion deviating in the placebo arm (31%). The distributions of the patterns of missingness between the two treatment groups do not differ (chi-squared test statistic D 5.15, *p*D0.161).

**Table 2:** Percentage of patients remaining in the study at each visit

	Placebo	Prednisolone
Month	N (%)	N (%)
0	64 (100)	73 (100)
0.5	64 (100)	73 (100)
1	57 (88)	63 (86)
3	53 (83)	51 (70)
6	44 (69)	46 (63)

**Table 3:** Monotone data: mean $\sqrt {\text {CD4}}$ count at each visit by dropout pattern and treatment arm

	Dropout time (months)					
Dropout pattern ^[a]^	0	0.5	1	3	6	N (%)
Placebo arm						
4	13.14	13.47	13.62	16.24	17.09	44 (69)
3	12.58	13.702	14.76	17.01	-	9 (14)
2	16.90	17.68	20.27	-	-	4 (6)
1	15.98	12.44	-	-	-	7 (11)
All patients mean (std)	13.61 (5.84)	13.65 (4.97)	14.26 (5.32)	16.37 (4.85)	17.09 (5.14)	64 (100)
Prednisolone arm						
4	13.18	15.52	14.70	16.76	16.82	46 (63)
3	19.89	19.27	19.51	21.17	-	5 (7)
2	9.04	12.26	12.49	-	-	12 (16)
1	11.64	11.43	-	-	-	10 (14)
All patients mean (std)	12.75 (5.10)	14.68 (5.71)	14.66 (5.84)	17.19 (4.80)	16.82 (4.72)	73 (100)

^a^Dropout patterns: 4 = subjects who had all measurements up to 6 months (completers), 3 = subjects who had measurements up to 3 months, 2 = subjects who had measurements up to 1 month, and 1 = subjects who had measurements up to 2 weeks

**Fig. 6:**
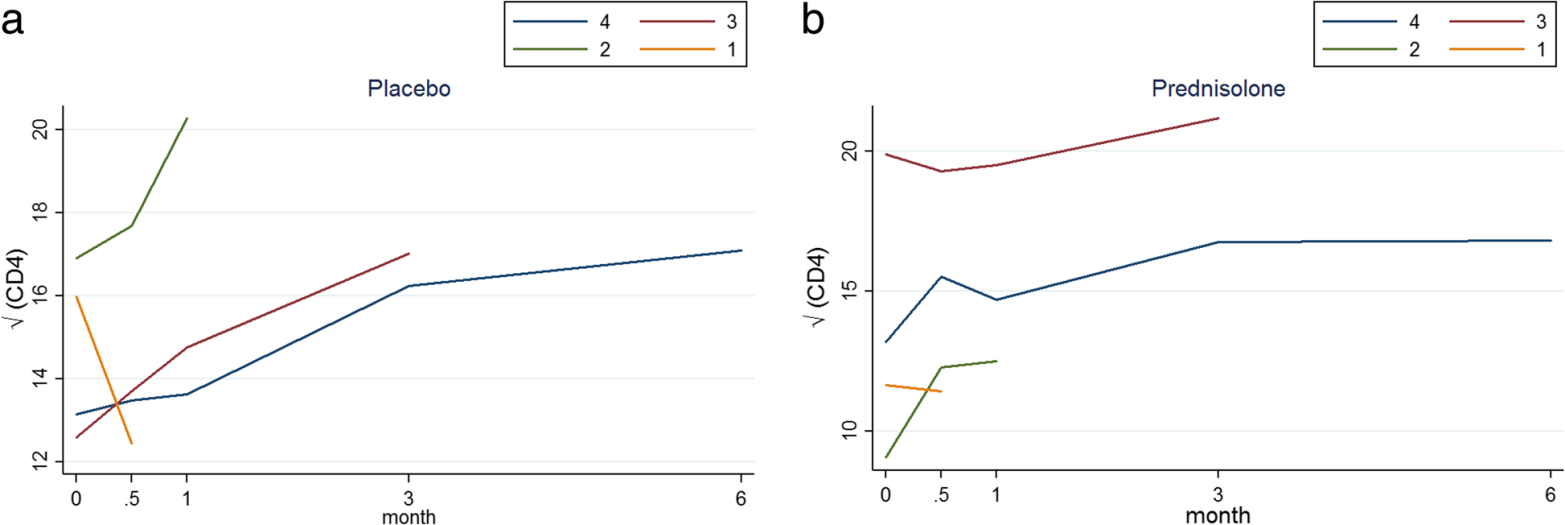
Profile plots of the mean of the $\sqrt {\text {CD4}}$ count for each deviation pattern under the placebo arm (left panel) and the active arm (right panel). Blue pattern: group of patients who completed the study (completers), brown pattern: group of patients who dropped out after month 3, green pattern: group of patients who dropped out after month 1 and yellow pattern: group of patients who dropped out after week 2

Figure 6 shows the profile plots of the mean $\sqrt {\text {CD4}}$ count of the four deviation patterns for patients in the placebo and prednisolone groups. This figure gives an indication that the $\sqrt {\text {CD4}}$ count increases over time. Figure 6 agrees with those mean profiles in Figs. 1 and 4. That is, there is slight increase in the $\sqrt {\text {CD4}}$ count among patients in the placebo arm compare with those in prednisolone arm.

## Estimands for primary and sensitivity analyses

Since the focus of this paper is to draw statistical inferences in the presence of missing data, this section discusses the *de jure* and *de facto* estimands [1]. This discussion is necessary because our primary analysis model is based on the *de jure* estimand, and the sensitivity analysis models are based on the *de facto* estimand [1]. The primary analysis (as specified in the statistical analysis plan) addresses the main objective of the study, whereas the sensitivity analysis considers models the make alternative assumptions (trial protocol) that, in one way or the other, may influence statistical inferences under the primary analysis model. We discuss the *de jure* estimand in “De jure estimand hypothesis” section and then the *de facto* estimand in “De facto estimand hypothesis” section. We will also discuss deviations associated with each estimand in “Deviations associated with estimands” section.

### De jure estimand hypothesis

The *de jure* estimand estimates the effect of treatment on patients assuming that patients adhered to the study protocol without deviating from the trial protocol [1, 14]. The *de jure* estimand hypothesis is analogous to the MAR mechanism. This hypothesis assumes that the conditional distribution of observations later in the follow-up, given observations earlier in the follow-up, is independent of whether deviation occurs. In this case, patients are expected to obtain the full benefit of the treatment and the question of interest is whether the treatment works under the best case scenario. In this study, the *de jure* primary analysis is based on the multiple imputation under missing at random (MAR) [8, 17, 20]. The primary analysis method to choose varies from trial to trial. The guidelines on how to decide on an appropriate primary analysis for a given trial can be found in the NRC panel report [3] and many others [14, 15].

### De facto estimand hypothesis

The *de facto* estimand concerns what would be the effect of treatment seen in practice if treatment were allocated to the target population of eligible patients as defined by the trial inclusion criteria. In addressing this question, we may ask, what would have been the effect of treatment seen at the end of the study if those who deviated moved to the equivalent of the active treatment arm (prednisolone treatment in this study). However, this may underestimate the benefit of active treatment in trials where more benefit is expected from the active treatment. This is because estimand equates treatment benefit of those failing on placebo arm to those opting for active treatment. In this instance, the fairer comparison might be to move those who deviate from the prednisolone arm onto the placebo arm. In the case of the IMPI trial, since all patients in both prednisolone and placebo arms were given ART, we expect no significant difference in their response to ART treatment unless there is interaction between prednisolone and ART treatment.

We discuss four *de facto* options for obtaining post-deviation data in “Constructing joint distributions of pre-deviation and post-deviation outcome data” section. These options make assumptions about the missing post-deviation data. These assumptions are alternative plausible assumptions, which depart from the MAR assumption under the primary analysis. In this way, it is assumed that the data are not missing at random (NMAR) and we assess the robustness of inferences under the MAR primary analysis to the alternative assumptions under the *de facto* options (sensitivity analyses).

### Deviations associated with estimands

It is important to define clearly deviations associated with each estimand in the study protocol. This is because clarity of deviations associated with each estimand is vital for primary analysis and framing relevant sensitivity analysis [1]. The exact definition of a deviation will depend on the trial setting and may also vary between separate analyses [1]. In the IMPI trial, the following situations can be regarded as deviations associated with the *de jure* estimand: unblinding of treatment arms and unobserved CD4 count measurements and deviations associated with the *de facto* estimand are unblinding such as treatment allocation, loss to follow-up such that no further treatment is taken and influence if trial prednisolone treatment on ART.

Given the estimands and their associated deviations, it is assumed that each patient has longitudinal follow-up data until either the patient deviates or reaches the final visit, and that the nature or reason of each deviation is known. This approach further assumes that for each deviation or group of similar deviations occurring in a dataset due to similar reasons, an appropriate post-deviation distribution can be built taking into consideration (1) the patient’s pre-deviations, (2) pre-deviations and post-deviations data from other patients in the trial, (3) the nature of the deviation, (4) and the reason for the deviation [1].

## Standard pattern-mixture model and the pattern-mixture model with multiple imputation

It this section, we give a brief review of the standard pattern-mixture model (PMM) and then discuss the pattern-mixture model with multiple imputation (PM-MI) of Carpenter and colleagues. In “Link between the pattern-mixture model and the pattern-mixture model with multiple imputation” section, we give the link between these approaches.

### Standard pattern-mixture model

We have mentioned in the “Background” section that the pattern-mixture modeling framework is a reverse factorization of the selection model [2, 8, 9, 21]. The selection model can be viewed as a multivariate model where one variable represents marginal density of the measurements process and the other variable represents the conditional density of the missingness process, given the outcomes. The PMM approach, on the other hand, is defined as a model for the product of the conditional distribution of the responses **Y**_*i*_ for patient *i*,*i*D1,2,…,*N*, given non-response patterns **R**_*i*_ and the model for non-response **R**_*i*_. [10, 22, 23]; that is

1 $$ \begin{aligned} \Pr \left(\mathbf{Y}_{i}, \mathbf{R}_{i} \mid \mathbf{X}_{i}, \boldsymbol{\theta}, \boldsymbol{\psi} \right) & = \Pr \left(\mathbf{Y}_{i} \mid \mathbf{X}_{i},\mathbf{R}_{i}, \boldsymbol{\theta}\right) \\ & \quad \times \Pr \left(\mathbf{R}_{i} \mid \mathbf{X}_{i}, \boldsymbol{\psi}\right), \end{aligned}  $$

where **R**_*i*_D1 if response is observed and 0 otherwise, **X**_*i*_ is design matrix of covariates, ***θ*** and ***ψ*** represent parameter estimates in the measurement model and dropout model respectively.

The PMM has desirable properties especially where the data are NMAR (probability that a response will be missing depends on the **R**_*i*_ and **Y**_*i*_). For instance, where it is not substantively reasonable to consider non-responses as missing data, it may be desirable to limit the inferences to the subpopulation of patients whose responses are observed. Thus, it is more meaningful to consider the distribution of **Y**_*i*_ given **R**_*i*_D1 (**R**_*i*_D1 if subject is observed and 0 otherwise) rather than the marginal distribution of *Y*_*ij*_ [8]. Contrary to the selection model, $\Pr \left (\mathbf {Y}^{m}_{i} \mid \mathbf {Y}^{o}_{i},\mathbf {X}_{i}, \mathbf {R}_{i} \right)$ is modeled directly from the pattern-mixture model, where $\mathbf {Y}^{o}_{i}$ is a vector of observed responses and $\mathbf {Y}^{m}_{i}$ is a vector of the missing responses.

One important feature of the pattern-mixture model (1) is that it fits a different response model for each pattern of missingness such that the observed data is a mixture of patterns weighted by their respective probabilities of missing patterns. That is, the first component in the PMM (1), Pr(**Y**_*i*_∣**X**_*i*_,**R**_*i*_,***θ***) fits a response model for each pattern of missingness and Pr(**R**_*i*_∣**X**_*i*_,***ψ***) represents dropout probability for each pattern. It follows that if there are *U* number of missingness patterns in a data set, then the marginal distribution of **Y**_*i*_ is a mixture of $\Pr \left (\mathbf {Y}_{i} \mid \mathbf {X}_{i}, \boldsymbol {\theta }\right) = \sum \limits _{u = 1}^{U} \Pr \left (\mathbf {Y}_{i} \mid \mathbf {R}_{i} = \mathbf {R}^{u}_{i}, \mathbf {X}_{i}, \boldsymbol {\theta }^{u}\right) \pi _{u}$, where *π*_*u*_DPr(**R**_*i*_D*u*∣**X**_*i*_,***ψ***) and **R**_*i*_ counts the number of *U* patterns, ***θ***^*u*^ represents the parameters of marginal density Pr(**Y**_*i*_) in the *u*^th^ pattern. It can be observed that in the pattern-mixture model, parameters {***θ***^1^,…,***θ***^*U*^} can have different dimensions. A logistic model is often assumed for dropout probabilities and a linear mixed effect model (LMM) [24] for the measurement process.

The linear mixed effects model (LMM) [24] is assumed for the measurement process and is given by

2 $$ \left\{ \begin{array}{l} \mathbf{Y}_{i} = \mathbf{X}_{i} \boldsymbol{\beta} + \mathbf{Z}_{i} \mathbf{b}_{i} + \boldsymbol{\epsilon}_{i},\\ \mathbf{b}_{i} \sim N\left(\boldsymbol{0}, \mathbf{G}_{i}(\boldsymbol{\rho})\right),\\ \boldsymbol{\epsilon}_{i} \sim N\left(\boldsymbol{0},\mathbf{R}_{i} (\boldsymbol{\sigma})\right),\\ \mathbf{b}_{i} \Perp \boldsymbol{\epsilon}_{i}, \end{array} \right.  $$

where **b**_*i*_ is an *q*-dimensional vector of random effects, **Z**_*i*_ and **X**_*i*_ are *N*×*q* and *N*×*q* dimensional matrices of known covariates, ***β*** is a *p*-dimensional vector containing the fixed effects, ***ε***_*i*_ is an *N*-dimensional vector of residual components, **G**_*i*_⊳***ρ***⊲ and **R**_*i*_⊳***σ***⊲ are *q*×*q* and *n*_*i*_×*n*_*i*_ covariance matrices respectively and ***σ*** and ***ρ*** are *c*×1 and *s*×1 (with *s*≤*n*_*i*_⊳*n*_*i*_C1⊲/2) vectors of unknown variance parameters corresponding to ***ε***_*i*_ and **b**_*i*_ respectively.

The pattern-mixture model (1) is well understood using the second MAR assumption. The second MAR assumption states that observations that would have been recorded for a patient in the future, given that the observed history of such patient has the same statistical behavior. This feature of the pattern-mixture model makes it possible for multiple imputation to provide a practical approach to estimation and inferences. In addition, this feature provides a framework for the formulation of the pattern-mixture model with multiple imputation [1].

### Pattern-mixture model with multiple imputation methodology

In this section, we describe the pattern-mixture model with multiple imputation (PM-MI) methodology [1]. Consider a randomized clinical trial with two treatment arms and predictors of continuous response **Y**_*i*_ (*Y*_*ij*_⊲ for each patient. Let the *Y*_*ij*_ be the measurements of the *i*^th^ patient at the *j*^th^ occasion in each treatment arm **T**_*i*_, where *j*D0 represents baseline measurements in each treatment arm and *j*D*n*_*i*_ denotes the last observation time prior to a deviation for the *i*^th^ patient. It is then assumed that all patients were observed at baseline. Let (1) $\mathbf {Y}^{o}_{i} = \left ({Y}_{i0}, \ldots, {Y}_{{in}_{i}} \right)'$ denotes a vector of the *i*^th^ patient’s observed responses at each scheduled visit from *j*D0,…,*n*_*i*_, (2) $\mathbf {Y}^{m}_{i} = \left ({Y}_{{in}_{i}+1}, \ldots, {Y}_{in} \right)'$ denote a vector of the *i*^th^ patient’s missing post-deviation responses at scheduled visits time from *j*D*n*_*i*_C1,…,*n*, where *n* is the last schedule visit, (3) $\mathbf {Y}^{m} = \left (\mathbf {Y}^{m'}_{1}, \ldots, \mathbf {Y}^{m'}_{N} \right)'$ denotes a column vector of the *i*^th^ patient’s missing post-deviation responses profile, and (4) $\mathbf {Y}^{o} = \left (\mathbf {Y}^{o'}_{1}, \ldots, \mathbf {Y}^{o'}_{N} \right)'$ denotes a column vector of the *i*^th^ patient’s observed responses profile. It follows that the distribution of each patient’s post-deviation responses $\mathbf {Y}^{m}_{i}$, given each patient’s pre-deviation responses $\mathbf {Y}^{o}_{i}$ and the deviation time *n*_*i*_, is defined by

3 $$ \Pr\left(\mathbf{Y}^{m}_{i} \mid \mathbf{Y}^{o}_{i}, n_{i}, \mathbf{T}_{i}, \boldsymbol{\theta}\right),  $$

where **T**_*i*_ denotes binary treatment arm (for patient in either the prednisolone or placebo treated arm). The parameter vector ***θ*** has to be estimated before we can impute missing post-deviation data by drawing from conditional distribution (3).

### Link between the pattern-mixture model and the pattern-mixture model with multiple imputation

If post-deviation data are assumed to be MAR (that is, the probability that the responses are missing depends on the observed data), the distribution (3) is independent of the deviation time *n*_*i*_. Hence the distribution (3) can be written as

4 $$ \Pr\left(\mathbf{Y}^{m}_{i} \mid \mathbf{Y}^{o}_{i}, \mathbf{T}_{i}, \boldsymbol{\theta}\right).  $$

Under such assumption, the direct maximum likelihood estimation [8, 25] or the multiple imputation under MAR can be used to obtain valid inference [8, 17, 26]. However, if data are NMAR, the distribution (3) depends on the deviation the time *n*_*i*_ in a manner that could be different for each patient. This feature of the distribution (3) is analogous to the standard pattern-mixture model (1), where response model is fitted for each pattern of missingness such that the observed data is a mixture of patterns weighted by their respective probabilities of missingness.

It follows that for each patient or group of patients, a specific form of the conditional distribution (3) is defined to reflect a specific assumption appropriate to their treatment arm **T**_*i*_, deviation time *n*_*i*_ and other relevant information or covariates. Given this information, multiple imputation is used for imputing missing post-deviation data from Eq. 3 to create complete data sets. Thereafter, estimation and inference is then performed by fitting a standard method of analysis (which is a methods of analysis that yields valid inferences without missing data) to the complete data sets [16, 17]. Thus, for inferences about ***θ*** in the presence of deviations, multiple imputation is used to create *K* “completed” data sets.

To obtain post-deviation data from the distribution (3), Carpenter and colleagues [1] suggested the following.

**Step A**: Assume a multivariate normal for the observed data **Y**^*o*^.

**Step B:** Draw samples of the parameter estimates of ***β*** and **R**_*i*_ from the Bayesian posterior distribution defined as Pr(***β***′,***α***′∣**Y**^*o*^), where ***β*** is a vector of the means and ***α***′D(***σ***′,***ρ***′)′ is a parameter vector of the variance components in the measurement model. The Markov chain Monte Carlo (MCMC) method is used to draw samples of ***β*** and ***α*** from this posterior.

**Step C:** Update the Markov chain sufficiently after each draw in order to avoid correlation between draws in each of the parameter estimates ***β*** and ***α***.

**Step D:** After each draw of ***β*** and ***α*** for each patient who deviates before the end of the trial, ***β*** and ***α*** are used to build the joint distribution of such patient’s pre-deviation and post-deviation data. We discuss different options for building this joint distribution in “Constructing joint distributions of pre-deviation and post-deviation outcome data” section.

**Step E:** The joint model in Step **D** is then used to build the conditional distribution of each patient’s missing post-deviation data, given the pre-deviation data (3). The missing post-deviation data in the conditional distribution (3) are obtained using the parameter estimates ***β*** and ***α*** obtained from **Step D**.

**Step F:** Repeat Steps **B-E***K* times to create *K* “complete” data sets. Thereafter, any method of analysis that yields valid inferences in the absence of missing data can then be applied to the complete data sets.

Carpenter and colleagues [1] considered the treatment benefit at the last schedule visit where they fitted a linear regression model that assumed that observations are independent. This paper considers the treatment benefit over time and hence the linear mixed effect model [24] is assumed for the measurement process. This model is then fitted to each of the *K* imputed data sets. This analysis produced *K* statistics for the parameter estimates ***β*** and ***α***. Estimates from each of the *K* completed data set were then combined to produce single estimates with their associated standard errors using the Rubin’s rule [17].

## Constructing joint distributions of pre-deviation and post-deviation outcome data

In this section, we discuss the four *de facto* options for obtaining the missing post-deviation data [1]. These options make alternative and plausible assumptions about the missing data such that the *de facto* (NMAR sensitivity analysis) assumptions depart from the *de jure* (MAR primary) assumption about the missing data. These assumptions assess whether inferences under such MAR primary analysis assumption are sensitive to the alternative plausible assumptions under NMAR sensitivity analysis. In this way, we will be able to assess whether the process that generated the missing CD4 count data is MAR or NMAR mechanism. This distinction is necessary because the type of missing data mechanism has implications for both the analysis and interpretations of the results [27]. We also discuss how to choose reference arm (“Choosing the reference arm” section) and the implications of the *de facto* options under the IMPI trial in “De facto options under the IMPI trial” section.

Carpenter and colleagues proposed the following options for constructing the joint distribution of each patient’s pre- and post-deviation outcome data where each option represents a possible *de jure* or *de facto* assumption concerning post-deviation data. These assumptions differ in the ways in which unavailable information for deviated patient are borrowed, or estimated, from other groups of patients in the same trial [1]. Here two treatment arms, placebo and active (prednisolone in our study), are considered and one of these arms is chosen as a reference arm such that unavailable information for deviated patient can be “borrowed” from such reference arm. The reference arm could be either the placebo or the active arm depending the hypothesis to address. In this study, we in turn used each arm as reference arm just to explore how treatment effect is affected under such considerations. Here, we refer to the arm not chosen as reference as the other arm.

**A**: *Jump to reference (J2R)*: Under this assumption, after a patient stops taking treatment from the randomized arm, such patient’s mean response distribution is now considered to be the same us of the “reference” group of patients. Typically, such a patient will take treatment from the control or placebo arm. However, such a patient may not necessarily take treatment from the placebo arm (but assumes to take treatment from the randomized arm after dropout) since the choice of the reference arm may depends on trial setting. In a trial where more benefit is expected in the active arm, such a change may be seen as extreme, and choosing the reference group to be the placebo group may be viewed as a worst-case scenario in terms of reducing any treatment benefit, since withdrawn patients on active will lose the effect of their period on treatment. In this study, the post-deviation data in the reference arm are imputed under randomized-arm MAR.

**B**: *Copy difference in reference (CDR)*: Under this *de facto* option, after the patient deviates, it is assumed that the patient’s post-deviation mean increments copy those from the reference arm. For instance, if the placebo arm is chosen as the reference arm, the patient’s mean profile after deviation tracks that of the mean profile in the placebo arm, but starting from the benefit already obtained from the active arm.

**C**: *Last mean carried forward (LMCF)*: Under the LMCF, it is assumed that after deviation, the patient’s post-deviation means equal that of the marginal mean of the randomized treatment arm.

**D**: *Copy reference (CR)*: The “copy reference” de fact option assumes that a patient’s whole distribution, both pre-deviation and post-deviation data, is the same as reference arm.

Whereas the above assumptions for constructing post deviation data have been proven to be practical and permit relevant, accessible assumptions for framing primary and sensitivity analyses, the PM-MI approach depends on the relevance of the assumptions about missing post-deviation data in relation to the context of the trial at hand [1]. In this study, we apply the PM-MI approach in the context of the IMPI trial setting (see “Choosing the reference arm” and “De facto options under the IMPI trial” sections).

### Choosing the reference arm

For the “jump to reference”, “copy reference” and “copy increment in reference” *de facto* options, we discuss the implications for the choice of the reference arm. In the IMPI trial, it could be either the placebo or the prednisolone arm. This is because we expect similar statistical behavior for patients in either arm. Suppose that one wishes to address the *de facto* question corresponding to the assumption that after post-deviation (CD4 count measurements are unobserved), (1) patients on the placebo arm obtain a treatment equivalent to the active (prednisolone) arm, and (2) the prednisolone-treated patients continue on treatment and adhered to the study protocol, so that their post-deviation data can be imputed assuming randomized-arm MAR. In such a case, we specify the prednisolone arm as a reference. In the IMPI trial, HIV+ patients in either placebo or prednisolone arm were given ART and thus patients with their CD4 count unobserved are expected to have equivalent treatment benefit compared with those patients with their CD4 count observed unless prednisolone treatment influences ART treatment. Since we hypothesized that patients’ response to ART treatment in both the placebo and the prednisolone arms are comparable, we also present results where the placebo arm is used as a “reference”. Thus dropouts in the prednisolone arms obtain treatment equivalent to the placebo arm so that their post-deviation data (unobserved CD4 count measurement) can be imputed under randomized-arm MAR. This latter assumption might be appropriate where no alternative treatment is generally available or where patients in both arms receive treatment but responses were unobserved (in the case of the IMPI trial IMPI trial).

### De facto options under the IMPI trial

A simple interpretation of the PM-MI approach is that within the same trial, the PM-MI approach is used to “borrow” or estimate unavailable information from a group of patients for another group of patients who have their information missing. As we have stated earlier, in the IMPI trial setting, HIV+ patients in both the active treatment (prednisolone) arm and the placebo treatment arm were given ART, and hence we expect similar benefit of ART treatment unless prednisolone treatment interacts with the ART treatment. One research question to address in the IMPI trial is whether the prednisolone treatment interacts with the ART treatment. If they do interact, patients’ response to ART treatment from the active arm and the placebo arm will be different, otherwise they would be comparable. Also in the IMPI trial, missing CD4 count for patients were unobserved due to inadequate resources but not necessarily that the patient dropped out before the end period of the trial. In other words, CD4 count measurements were missing at some scheduled visits mostly due to administrative reason and missingness would have been generated by a random process. In fact, only 6% of the patients dropped out (genuine dropout) in the IMPI trial. This means that most of the patients do not dropout from the study but their CD4 count values could not be measured due to inadequate resources. Thus, patients who CD4 count are unobserved, are expected to have similar CD4 count levels to those who were observed. Out of a total number of 294 HIV positive patients in the placebo arm, approximately 78% were already on ART at the time of randomization and out of a total number of 293 HIV positive patients in the prednisolone arm, approximately 80% were already on ART at baseline.

For the *de facto* question, since we do not expect significant different in treatment effect between patients with their CD4 count observed and those with their CD4 count unobserved, the jump to reference and the copy reference options are the most plausible options for assessing sensitivity of inferences to MAR assumption.

The CD4 count data introduced in “Description of the IMPI trial data” section, are analyzed under *de jure* MAR and *de facto* NMAR assumptions. In the measurement model (2), we included an intercept, and assumed as fixed effects the following covariates: prednisolone (which takes the value of 1 for subjects randomized to prednisolone and 0 if the subject was randomized to placebo), time (months), age, whether on ART or not at each scheduled visit (1 if the subject received ART, and 0 if subject did not receive ART), and interactions between prednisolone and time, and prednisolone and ART. Age and time were continuous variables. Our fitted linear mixed model is defined as

5 $$ \begin{aligned} {}\sqrt{\text{CD4}_{ij }} =& \beta_{0} + \beta_{1} \times \text{prednisolone}_{i} + \beta_{2} \times \text{month}_{j} \\ & + \beta_{3}\times\text{prednisolone} \times \text{month}_{ij} + \beta_{4}\times\text{ART}_{ij} \\ & + \beta_{5}\times\text{prednisolone} \times \text{ART}_{ij} + \beta_{6}\times\text{Age}_{i} \\ & + \mathrm{b}_{i} + {\epsilon}_{ij}, \end{aligned}\vspace*{-5pt}  $$

where $\sqrt {\text {CD4}_{ij}}$ is the square root of CD4 count for *i*^th^ patient at the *j*^th^ visit, for *i*D1,…,*N* and *j*D1,…,*n*_*i*_, b_*i*_ represents the patient-specific random effect, and *ε*_*ij*_ is the residual error. It is assumed that b_*i*_ and *ε*_*ij*_ are independently distributed as $\mathrm {b}_{i} \sim N\left (0,\sigma ^{2}_{b}\right)$ and ${\epsilon }_{ij} \sim N\left (0,\sigma ^{2}_{\epsilon }\right)$ respectively.

## Application of the PM-MI approach to the IMPI trial CD4 count data

In this section, we applied the PM-MI approach to the incomplete CD4 count data. We implemented the PM-MI approach using STATA **mimix** package developed by Cro of London School of Hygiene and Tropical Medicine (LHTM), UK. This package imputes missing continuous outcomes for a longitudinal trial with protocol deviations under distinct reference groups based assumptions for the unobserved data, following the procedure proposed by Carpenter and colleagues [1].

To address the *de jure* hypothesis, we performed multiple imputation for the unobserved CD4 count under MAR mechanism using the **ice** package in STATA [28]. We also impute post-deviation under LMCF, J2R, CDR and CR *de facto* options to obtain a complete data sets. The linear mixed effect model (5) was then fitted to each of the completed data sets and parameter estimates combined to produce parameter estimates with their corresponding standard errors using the Rubin’s rule [17, 28].

### Monotone data

**Fig. 7:**
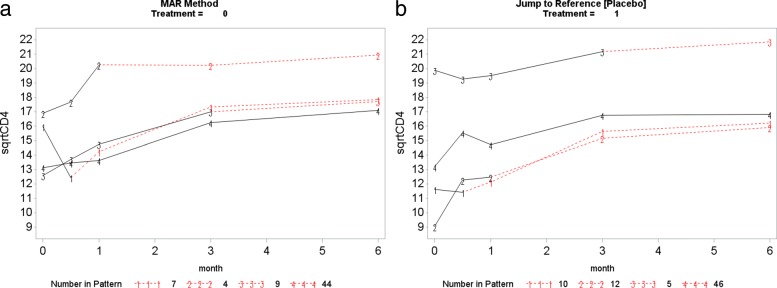
Placebo reference arm (Treatment = 0): Profile plots of the mean $\sqrt {\text {CD4}}$ count against month for the four different deviation patterns. The solid lines join the observed means (before deviation) and the dotted lines join the means of the imputed data for that pattern. Pattern 4: group of patients who completed the study (completers), Pattern 3: group of patients who dropped out after month 3, Pattern 2: group of patients who dropped out after month 1 and Pattern 1: group of patients who dropped out after week 2

**Fig. 8:**
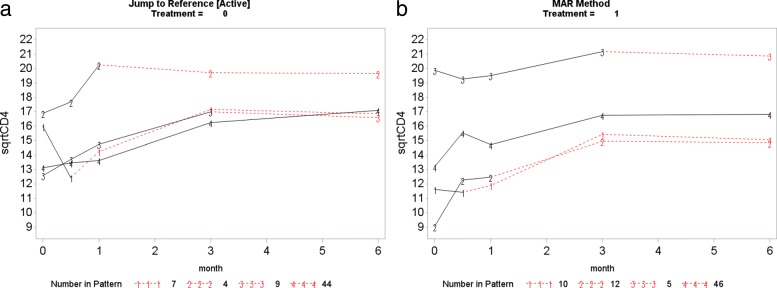
Prednisolone reference arm (Treatment = 1): Profile plots of the mean $\sqrt {\text {CD4}}$ count against month for the four different deviation patterns. The solid lines join the observed means (before deviation) and the dotted lines join the means of the imputed data for that pattern. Pattern 4: group of patients who completed the study (completers), Pattern 3: group of patients who dropped out after month 3, Pattern 2: group of patients who dropped out after month 1 and Pattern 1: group of patients who dropped out after week 2

This section presents the PM-MI analyses of the monotone CD4 count data. We consider the jump to reference option for illustration purpose and Fig. 7 shows profiles plots of the mean $\sqrt {\text {CD4}}$ count measurements for the complete data sets, for each deviation pattern, by placebo arm (Treatment = 0) and prednisolone arm (Treatment = 1). The left panel of the Fig. 7 shows complete data profiles of the placebo reference arm with missing post-deviation values obtained under MAR whereas the right panel of the Fig. 7 shows complete data profiles of the prednisolone arm patients with missing post-deviation data “borrowed” from the placebo arm (left panel of the Fig. 7). We in turn used the prednisolone arm as a reference where the complete data profiles are shown in Fig. 8. The right panel of the Fig. 8 shows complete data profiles of the prednisolone reference arm with missing post-deviation values obtained under MAR whereas the left panel of the Fig. 8 shows complete data profiles of the placebo arm patients with missing post-deviation data obtained from the prednisolone arm (right panel of the Fig. 8). It can be observed that treatment seems to reduce CD4 count a little, and so imputed data for placebo under MAR are above those when the placebo patient jumps to the prednisolone arm. Hence, we investigate the significance of such reduction in the CD4 count level by using the parameter estimates associated with the prednisolone-ART interaction (see Table 4). Similar plots for LMCF, CDR and CR can be found in Appendix A. After imputation of the missing post-deviation data under LMCF, J2R, CDR and CR, we fit a linear mixed effect model (5) to the completed data sets and combine the parameter estimates from each data set using the Rubin’s rule to produce parameter estimates with their associated standard errors for the final inferences. The parameter estimates from these analyses are shown in Table 4.

**Table 4:** Monotone data: Parameter estimates from *de jure* and *de facto* analyses

	Prednisolone			Time			Prednisolone x Time			ART			Prednisolone x ART			Age		
Analysis	Est	s.e.	*p*-value	Est	s.e.	*p*-value	Est.	s.e.	*p*-value	Est.	s.e.	*p*-value	Est.	s.e.	*p*-value	Est.	s.e.	*p*-value
MI	0.56	0.791	0.481	0.39	0.140	0.005	0.13	0.195	0.494	2.76	0.689	<0.0001	-1.12	0.966	0.245	-3.12	1.372	0.023
LMCF	0.46	0.870	0.597	0.34	0.092	<0.0001	-0.05	0.146	0.751	2.81	0.548	<0.0001	-0.75	0.875	0.400	-3.06	1.529	0.046
J2R (P-)	0.42	0.845	0.619	0.53	0.094	<0.0001	0.04	0.161	0.825	2.33	0.556	<0.0001	-0.96	0.842	0.260	-3.00	1.509	0.047
J2R (P+)	0.42	0.849	0.623	0.42	0.103	<0.0001	0.02	0.130	0.879	2.57	0.544	<0.0001	-0.96	0.786	0.275	-3.00	1.518	0.048
CDR (P-)	0.46	0.851	0.586	0.52	0.094	<0.0001	0.05	0.148	0.729	2.33	0.56	<0.0001	-0.96	0.844	0.259	-2.97	1.507	0.049
CDR (P+)	0.46	0.863	0.593	0.41	0.113	0.001	0.027	0.147	0.852	2.59	0.589	<0.0001	-0.88	0.882	0.3230	-2.98	1.52	0.050
CR (P-)	0.43	0.852	0.613	0.53	0.093	<0.0001	0.002	0.144	0.990	2.34	0.554	<0.0001	-0.87	0.815	0.288	-3.00	1.509	0.047
CR (P+)	0.44	0.858	0.608	0.43	0.114	<0.0001	0.006	0.137	0.966	2.55	0.579	<0.0001	-0.839	0.848	0.328	-3.00	1.521	0.049

Table 4 shows inferences from the MAR primary analysis (MI), which addresses the *de jure* hypothesis, are robust to the difference assumptions under the NMAR sensitivity analyses under *de facto* estimand hypothesis (LMCF, J2R, CR, and CDR). This result thus serves as a justification that the mechanism that generated the missing data in the CD4 count measurements from the IMPI trial is missing at random (MAR) mechanism. The implication of this justification is that the direct maximum likelihood and multiplication methods under MAR can be used to provide valid inferences when assessing the effect of prednisolone and ART treatments on changes in CD4 count level among different treatment groups. The results show that there is no significant prednisolone effect. The effect of prednisolone-ART is also not significant. This confirmed our hypothesis that prednisolone treatment does not influence ART treatment. However, there seem to be a slight reduction of CD4 count level in the prednisolone arm. Patients’ CD4 count levels increased significantly with time and patients who are permanently on ART have significantly higher CD4 count levels relative to those who are not ever on ART treatment. The prednisolone-time interaction results show a very slight increase in CD4 count level in the placebo arm compared with prednisolone arm over time. However, this increase is not significant. The near-zero estimates of the prednisolone-time interaction effect suggest that there is no difference in prednisolone effect in both arms over time. This means that the effect of treatments in both arms does not differ significantly over time. The results also show that older patients are more likely to have lower CD4 count, hence CD4 count significantly decrease with increasing age. These results agree with the mean $\sqrt {\text {CD4}}$ count profiles plots in Figs. 1 and 4. This is because CD4 count in both the prednisolone and placebo arms increases at the same rate (no significant prednisolone effect and prednisolone does not influence ART treatment) and CD4 count increases with increasing time where this increase, in both arms, is the same over time (no prednisolone-time effect).

### Combined monotone and non-monotone data

This section presents the PM-MI analyses of the combined monotone and non-monotone data. Parameter estimates of these analyses are shown in Table 5. The results of these analyses agree with the results under the Table 4. These results also give an indication that the MAR primary analysis (MI), which addresses the *de jure* hypothesis, are robust to the difference assumptions by and the NMAR sensitivity analyses under *de facto* estimand hypothesis (LMCF, J2R, CR, and CDR). These analyses show that the mechanism that generated the missing data in the CD4 count measurements from the IMPI trial is missing at random (MAR) mechanism. This means that the direct maximum likelihood and multiplication methods under MAR can be used to provide valid inferences when assessing the effect of prednisolone and ART treatments on changes in CD4 count level among different treatment groups.

**Table 5:** Combined non-monotone and monotone data: Parameter estimates from *de jure* and *de facto* analyses

	Prednisolone			Time			Prednisolone x Time			ART			Prednisolone x ART			Age		
Analysis	Est	s.e.	*p*-value	Est	s.e.	*p*-value	Est.	s.e.	*p*-value	Est.	s.e.	*p*-value	Est.	s.e.	*p*-value	Est.	s.e.	*p*-value
MI	-0.61	0.42	0.150	0.51	0.079	<0.0001	-0.004	0.115	0.974	1.50	0.394	<0.0001	0.21	0.581	0.718	-2.51	0.667	<0.0001
LMCF	-0.76	0.446	0.088	0.39	0.045	<0.0001	0.03	0.081	0.707	2.21	0.263	<0.0001	-0.04	0.406	0.924	-2.29	0.799	0.004
J2R (P-)	-0.36	0.486	0.455	0.47	0.047	<0.0001	0.04	0.076	0.561	2.03	0.258	<0.0001	-0.30	0.404	0.457	-2.25	0.779	0.004
J2R (P+)	-0.36	0.512	0.489	.45	0.052	<0.0001	0.03	0.080	0.737	2.06	0.321	<0.0001	-0.16	0.438	0.712	-2.25	0.795	0.001
CDR (P-)	-0.61	0.444	0.168	0.47	0.047	<0.0001	.05	0.083	0.577	2.04	0.259	<0.0001	-0.21	0.379	0.582	-2.27	0.783	0.0004
CDR (P+)	-0.59	0.471	0.216	0.46	0.051	<0.0001	0.02	0.083	0.849	1.93	0.288	<0.0001	-0.03	0.397	0.939	-2.24	0.790	0.005
CR (P-)	-0.64	0.438	0.143	0.47	0.047	<0.0001	0.04	0.075	0.644	2.04	0.258	<0.0001	-0.15	0.376	0.688	-2.29	0.782	0.003
CR (P+)	-0.66	0.451	0.142	0.46	0.050	<0.0001	0.02	0.079	0.792	1.91	0.288	<0.0001	-0.01	0.405	0.979	-2.22	0.792	0.001

It can be observed from these analyses that there is no significant prednisolone effect and the effect of prednisolone-ART is also not significant. This implies that prednisolone treatment does not influence ART treatment. We also found a reduction of CD4 count level in the prednisolone arm. However, this reduction is not significant. As expected, patients’ CD4 count levels significantly increase with increasing time and patients who are on ART at each schedule visit time have significantly higher CD4 count levels relative to those who are not on ART treatment at each schedule visit. The near zero estimates of the prednisolone-time interaction effect suggest that there is no difference in prednisolone effect in both arms over time. This means that the effect of treatments in both arms does not differ significantly over time. The results also show that older patients are more likely to have lower CD4 values, hence CD4 count significantly decrease with increasing age. These results agree with the mean $\sqrt {\text {CD4}}$ count profiles plots in Figs. 1 and 4. This is because CD4 count in both the prednisolone and placebo arms increases at the same rate (no significant prednisolone effect and prednisolone does not influence ART treatment) and CD4 count increases with increasing time where this increase, in both arms, is the same over time (no prednisolone-time effect).

## Simulation study

In this section we performed simulation experiments to evaluate the performance of the PM-MI approach. We performed a simulation experiment to evaluate the performance of the *de facto* hypothesis against the usual MI method for imputation of missing data and likelihood based method (ML). These methods (MI and ML) are known to provide valid inference when missing values are missing at random (MAR) [8, 17].

The simulated datasets were generated using the R software. The R code for the simulation experiment is available from the first author upon request. The simulation experiment was performed according to the linear mixed effect model defined by

$$ \begin{aligned} \mathrm{Y}_{ij} =& \beta_{0} + \beta_{1} \times \text{treatment}_{i} + \beta_{2}\times\text{time}_{j} \\ & + \beta_{3} \times \text{treatment} \times \text{time}_{ij} + \mathrm{b}_{i} + {\epsilon}_{ij}. \end{aligned} $$

The initial values for *β*_0_,*β*_1_,*β*_2_, and *β*_3_ are 13, 0.75, 0.11, -0.19, 0.20 respectively. The initial value for standard deviation *σ* of the random effect b_*i*_ is 4.57. In generating these data sets, we assumed that (1) the measurement at the first time point (*j* = 0) from the original data set is completely observed, (2) the data are MCAR or MAR mechanism, (3) the missing pattern is monotone, and (4) there are different dropout rates. We considered the following two steps for generating the data sets. We called these steps, M-step and D-step. We generated the longitudinal measurements under the M-step and under the D-step, we then generated data according to MAR and MCAR mechanisms.

**M-step**: We generated five-repeated measurements for each patient by a random number from a multivariate normal distribution. We used parameter estimates obtained from fitting a linear mixed effect model to the data. We repeated these processes 1000 times for 200 patients. Patients were randomly assigned to two treatment (treatment and placebo) arms in a ratio 1:1.

**D-step**: We generated missing data according to MCAR and MAR mechanisms. Missing data were generated through a logistic regression model. However, generating MCAR and MAR missing mechanisms involves two different assumptions for the dropout mechanism. For MAR, missing data were generated by dropping observations according to a logistic regression model relating the probability of dropout at particular time point with changes from baseline to previous time point. For MCAR, missing data were randomly generated by dropping observations according to a logistic regression model. Specific values for the logistic regression were chosen in order to yield the desire dropout rates in a given missing data mechanism. Under each of the missing data mechanisms, we generate overall dropout rates at 5%, 20%, 30, and then 50%. Thereafter, we perform analyses using ML, MI, LMCF, J2R, CDR and CR approaches and then assess the performance of these methods in estimating treatment effect.

The results from the simulation study under MCAR and MAR mechanisms are shown in Appendix B. The MCAR results are shown in Table 6 and the MAR results are presented in Table 7. Under the MCAR mechanism, it can be observed that all the methods produced unbiased parameter estimates under the different missingness rates. The root mean square error (RMSE) estimates of prednisolone effect, produced by each methods under the different missingness rates, are often higher compared with the time and treatment-time interaction effects. Most of the methods yielded unbiased estimates of treatment effect and this may imply that the process that generated the missing data is likely to be random. The simulation results under the MAR mechanism revealed that each of the methods yielded unbiased estimates for prednisolone effect under the missingness rates with less unbiased estimates for treatment effects when the missingness rates are 5%, 20% and 50%. All the methods yielded unbiased estimate of time effect under the different missingness rates. When missingness rate was assumed to be 50%, the LMCF and the CDR methods yielded less unbiased estimates of time effect. Each of the methods showed no bias for treatment-time interaction slope when the missingness rates were assumed to be 5%, 10% and 30% and bias for treatment-time interaction slope when missingness rate was assumed to be 50% and 20%. However, ML and MI, yielded unbiased estimates for treatment-time interaction. These results suggested that the four *de facto* assumptions proposed by Carpenter and colleagues [1] are suitable for handling the missing data in the IMPI clinical trial and other trials with similar settings.

## Discussion and conclusion

In this paper, we investigated the effect of TB pericarditis treatment (prednisolone) on CD4 count changes over time. We also conducted sensitivity analysis to investigate sensitivity of statistical inferences under MAR analysis (*de jure* option) to alternative plausible assumptions under NMAR (*de facto* option) using the PM-MI approach [1]. These principles and methods quantify the robustness of inferences to departures from the primary analysis assumptions. We recognized that this case study cannot cover the broad range of types and designs of clinical trials. This is because the literature on sensitivity analysis is evolving. The primary objective of this paper is to assert the importance of conducting some form of sensitivity analysis and to illustrate principles in the IMPI trial setting.

**Fig. 9:**
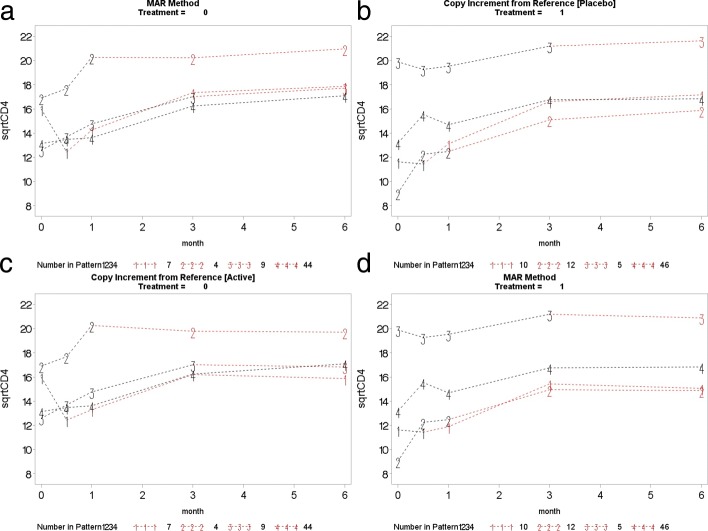
Copy increment from reference (CDR): placebo arm (top left panel) used as reference to impute data for the active arm (top right panel). Active arm (bottom right panel) used as reference to impute data for the placebo arm (bottom left panel)

**Fig. 10:**
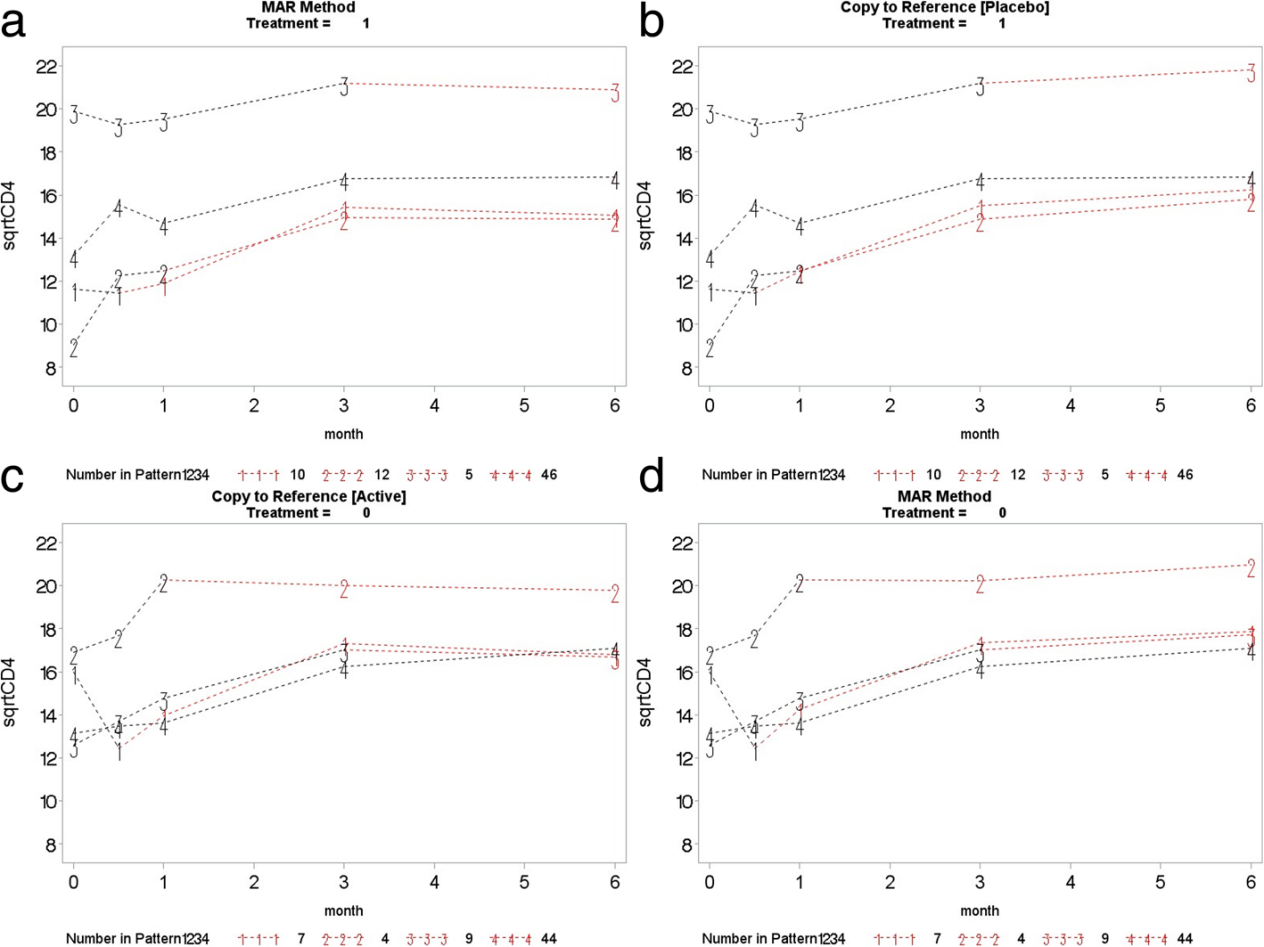
Copy to reference (CR): placebo arm (top left panel) used as reference to impute data for the active arm (top right panel). Active arm (bottom right panel) used as reference to impute data for the placebo arm (bottom left panel)

**Fig. 11:**
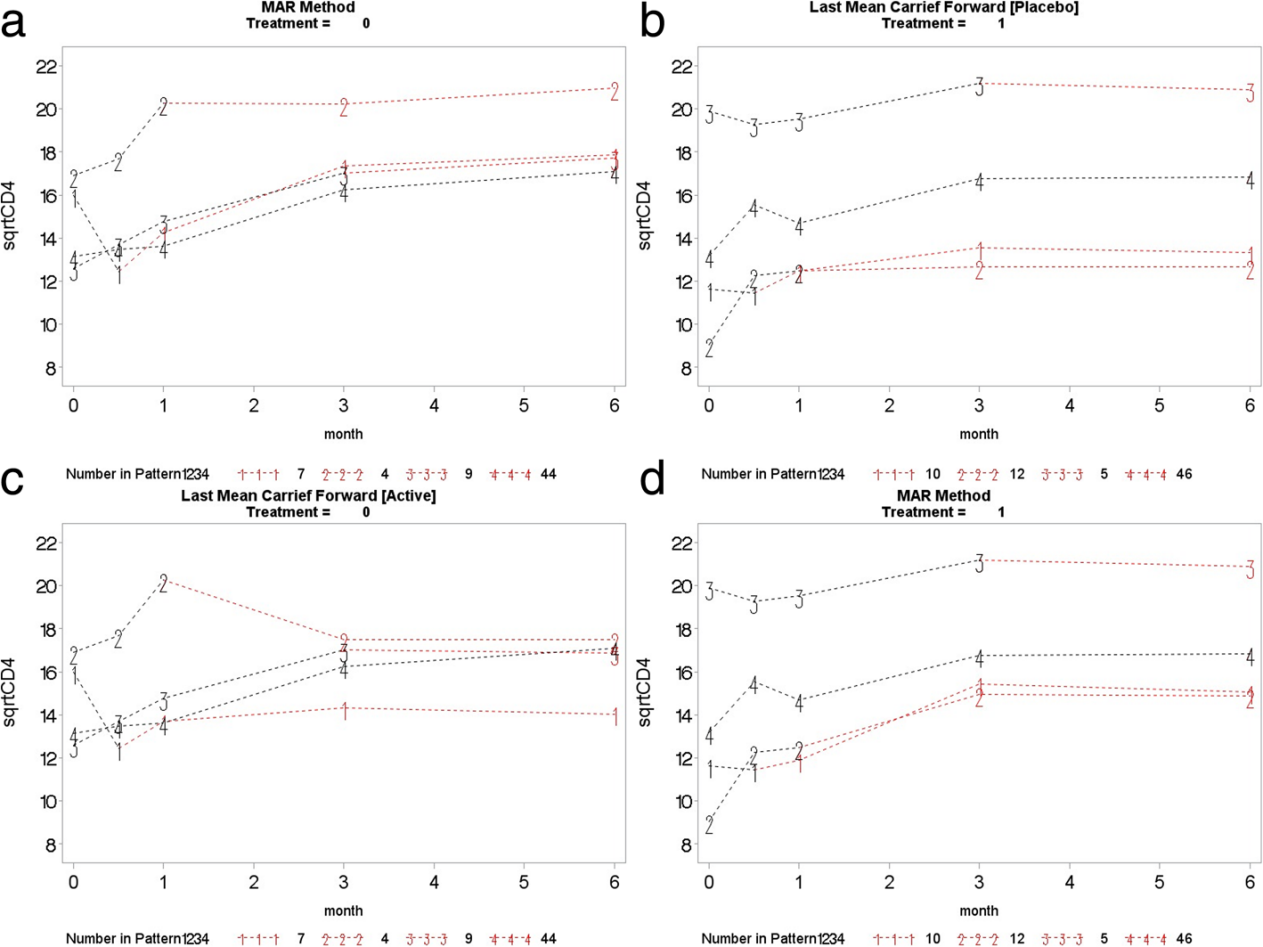
Last mean carried forward (LMCF): placebo arm (top left panel) used as reference to impute data for the active arm (top right panel). Active arm (bottom right panel) used as reference to impute data for the placebo arm (bottom left panel)

**Table 6:** MCAR mechanism by missingness rate

Missingness rates		Treatment		Time		Treatment x Time		Coverage probability		
5%	Analysis	Bias	RMSE	Bias	RMSE	Bias	RMSE	Treatment	Time	Treatment x Time
	ML	-0.063	0.127	-0.005	0.015	0.028	0.033	94.10%	96.40%	93.50%
	MI	-0.0602	0.127	0.001	0.020	0.021	0.031	95.50%	94.10%	95.80%
	LMCF	-0.079	0.137	-0.049	0.051	0.032	0.039	93.50%	95.00%	95.60%
	J2R (P-)	-0.100	0.149	-0.006	0.016	0.050	0.054	92.70%	95.40%	94.30%
	CDR (P-)	-0.090	0.141	-0.006	0.016	0.040	0.046	93.10%	95.30%	94.20%
	CR (P-)	-0.095	0.146	-0.006	0.016	0.045	0.050	92.20%	95.30%	94.80%
10%										
	ML	0.112	0.157	-0.017	0,022	0.028	0.035	89.50%	94.50%	95.00%
	MI	0.112	0.158	-0.018	0.024	0.030	0.040	90.50%	95.50%	95.00%
	LMCF	0.095	0.150	-0.103	0.104	0.040	0.046	92.50%	87.50%	95.50%
	J2R (P-)	0.072	0.133	-0.015	0.020	0.054	0.058	94.5%	87.50%	94.50%
	CDR (P-)	0.088	0.143	-0.015	0.020	0.046	0.051	92.50%	86.50%	96.00%
	CR (P-)	0.078	0.136	-0.015	0.020	0.052	0.057	93.50%	88.50%	95.50%
20%										
	ML	-0.032	0.12	-0.0024	0.017	0.024	0.034	95.00%	94.50%	95.00%
	MI	-0.042	0.124	-0.005	0.022	0.033	0.045	94.50%	95.50%	95.00%
	LMCF	-0.099	0.154	-0.183	0.183	0.078	0.083	93.50%	90.50% %	94.50%
	J2R (P-)	-0.122	0.164	0.0002	0.016	0.092	0.100	89.50%	95.00%	94.50%
	CDR (P-)	-0.074	0.134	0.0002	0.016	0.057	0.063	95.50%	95.00%	93.50%
	CR (P-)	-0.099	0.149	0.0002	0.016	0.073	0.075	94.50%	95.00%	94.50%
30%										
	ML	-0.092	0.149	-0.052	0.055	0.043	0.051	94.30%	94.50%	95.10%
	MI	-0.050	136	-0.030	0.039	0.005	0.037	94.40%	95.20%	95.80%
	LMCF	-0.136	0.184	-0.279	0.280	0.081	0.086	89.10%	85.70%	90.20%
	J2R (P-)	-0.220	0.252	-0.059	0.062	0.138	0.141	85.20%	94.50%	87.40%
	CDR (P-)	-0.160	0.200	-0.059	0.062	0.094	0.098	90.50%	94.10%	95.30%
	CR (P-)	-0.197	0.216	-0.059	0.062	0.109	0.112	90.90%	93.20%	92.70%
50%										
	ML	0.036	0.123	0.0004	0.022	0.009	0.033	96.60%	95.30%	95.40%
	MI	0.039	0.136	-0.006	0.031	0.026	0.051	95.30%	95.10%	94.60%
	LMCF	-0.045	0.129	-0.360	0.360	0.084	0.088	94.30%	89.10%	95.40%
	J2R (P-)	-0.112	0.159	0.0004	0.023	0.127	0.130	89.50%	95.10%	90.60%
	CDR (P-)	-0.045	0.121	0.0004	0.023	0.088	0.089	94.70%	95.20%	93.40%
	CR (P-)	-0.087	0.142	0.0004	0.023	0.112	0. 115	91.20%	95.20%	89.50%

**Table 7:** MAR mechanism by missing rate

Missingness rates		Treatment		Time		Treatment x Time		Coverage probability		
5%	Analysis	Bias	RMSE	Bias	RMSE	Bias	RMSE	Treatment	Time	Treatment x Time
	ML	-0.153	0.189	-0.0112	0.018	0.009	0.022	87.50%	95.30%	95.10%
	MI	-0.155	0.191	-0.008	0.017	0.004	0.022	86.90%	95.50%	94.50%
	LMCF	-0.155	0.190	-0.033	0.036	0.011	0.023	85.90%	95.10%	95.30%
	J2R (P-)	-0.172	0.205	-0.012	0.019	0.023	0.031	82.50%	94.90%	95.20%
	CDR (P-)	-0.160	0.195	-0.012	0.019	0.015	0.025	87.30%	95.10%	94.50%
	CR (P-)	-0.165	0.199	-0.012	0.018	0.019	0. 027	83.70%	94.30%	95.50%
10%										
	ML	0.062	0.125	0.003	0.015	0.011	0.024	94.50%	95.10%	94.20%
	MI	0.059	0.125	-0.007	0.017	0.016	0.030	94.20%	95.10%	94.30%
	LMCF	0.031	0.11	-0.087	0.088	0.034	0.040	95.10%	93.30%	95.20%
	J2R (P-)	0.013	0.110	-0.004	0.016	0.044	0.050	94.50%	95.20%	96.60%
	CDR (P-)	0.036	0.115	-0.004	0.016	0.032	0.040	95.69%	95.90%	95.20%
	CR (P-)	0.022	0.111	-0.004	0.016	0.040	0. 045	95.30%	95.10%	95.60%
20%										
ML	-0.043	0.012	-0.0061	0.018	0.009	0.025	94.10%	94.50%	95.30%	
	MI	-0.036	0.125	-0.013	0.026	0.013	0.033	95.00%	94.50%	95.30%
	LMCF	-0.127	0.175	-0.180	0.180	0.066	0.070	87.90%	84.40%	94.50%
	J2R (P-)	-0.131	0.177	-0.002	0.017	0.078	0.083	86.80%	95.30%	95.10%
	CDR (P-)	-0.102	0.155	-0.002	0.017	0.054	0.059	85.30%	96.10%	95.50%
	CR (P-)	-0.115	0.163	-0.002	0.017	0.063	0. 067	89.60%	95.10%	95.40%
30%										
	ML	0.062	0.125	0.003	0.015	0.011	0.024	94.50%	95.40%	96.30%
	MI	0.059	0.125	-0.007	0.017	0.016	0.030	95.10%	94.50%	96.20%
	LMCF	0.031	0.11	-0.087	0.088	0.034	0.040	94.30%	95.50%	94.70%
	J2R (P-)	0.013	0.110	-0.004	0.016	0.044	0.050	94.50%	96.10%	95.90%
	CDR (P-)	0.036	0.115	-0.004	0.016	0.032	0.040	95.10%	94.30%	95.50%
	CR (P-)	0.022	0.111	-0.004	0.016	0.040	0.045	94.50%	95.60%	96.20%
50%										
	ML	0.153	0.193	0.055	0.059	-0.071	0.077	87.80%	94.50%	96.10%
	MI	0.144	0.195	0.050	0.059	-0.063	0.077	86.30%	95.40%	96.10%
	LMCF	0.066	0.143	0.374	0.374	0.090	0.094	93.10%	67.80%	92.50%
	J2R (P-)	0.142	0.185	0.054	0.060	0.122	0.125	87.70%	96.10%	89.50%
	CDR (P-)	-0.039	0.124	0.054	0.060	0.056	0.061	95.50%	94.80%	96.40%
	CR (P-)	-0.102	0.156	0.54	0.060	0.095	0.098	93.10%	92.70%	95.40%

The study results show that inferences under the *de jure* (MAR primary analysis) assumption are robust to the inferences under the *de facto* (NMAR sensitivity analysis) assumptions. This finding gives an indication that the mechanism that generated the missing values in the CD4 count measurements from the IMPI trial is likely to be missing at random (MAR). The implications are that (1) the observed data are random sample from the population patients with TB pericarditis and (2) either the direct maximum likelihood (ML) approach or the multiple imputation approach, under the assumption that the data are MAR, can be used to produce valid inferences.

The investigation of sensitivity of statistical inferences to missing data is important and use of such methods must be encouraged. This is because, such sensitivity analysis provides additional information to readers of a clinical report to be able to interpret the results. This means that clinical reports should describe the primary and the sensitivity analyses to non-statisticians. This requires that assumptions about missing data are articulated in a transparent manner so that researchers and practicing clinicians can assess their validity under the study at hand [1]. Carpenter and colleagues [1] encourage the need for such sensitivity analysis stating that “assumptions need to be assessable, so that in the context of the trial at hand all stakeholders can understand whether they are plausible. Then, departures from these assumptions also need to be relevant in the context of the trial at hand, so that stakeholders can see if they require investigation.” When data are missing, it is possible that readers of a clinical report may doubt its conclusions unless the conclusions are supported with sensitivity analysis.

Our study results from both the combined monotone, and the non-monotone and monotone showed that there is no significant prednisolone effect in all the analyses. The prednisolone-time interaction results show a very slight reduction in CD4 count level among the patients in the prednisolone arm compared with placebo arm over time. However, this reduction is not significant. As expected, there is a significant time effect indicating that CD4 count level increases with increasing time. Patients who are on ART treatment, at each scheduled visit, are likely to have significantly higher CD4 count levels compared with those who are not always on ART at each visit time. The results also show that older patients are more likely to have a lower CD4 count level. Also, there is no prednisolone-ART interaction effect in all the analyses. However, the prednisolone effects under the combined monotone and non-monotone analyses are negatives because the overall reduction in the CD4 count levels among patients in the prednisolone arm is more pronounced than that of the patients in the placebo arm (see Fig. 1). On the contrary, the treatment effects under the non-monotone analyses are positives because the overall reduction in the CD4 count levels among patients in the prednisolone arm is less pronounced than that of the patients in the placebo arm (see Fig. 4).

The IMPI trial was a cardiology trial and HIV-related data were collected. However, the HIV data were not collected as would have be in a HIV focused clinical trial, and hence there are missing CD4 count. Despite the fact that the IMPI trial is a cardiology trial, our analyses of the HIV data provide reasonable information regarding the effect of prednisolone on CD4 count changes over time.

In the IMPI trial prednisolone effect was not significant, and hence patients CD4 count levels in the treatments arms are comparable. If the prednisolone effect was significant, CD4 count levels for patients in the treatment arms would have been different.

The missingness of CD4 values might be informative, and hence later values of CD4 count might be missing because patients died. This would require joint modeling on the CD4 count and time to death.

## Appendix A

This section presents the complete profile plots of **CDR**, **CR**, and **LMCF***de facto* hypotheses.

## Appendix B

This section presents simulation results under MCAR and MAR mechanisms with varying missingness rates 5%, 10%, 20%, 30%, and 50% in Tables 6 and 7 respectively.

## Abbreviations

ART: Anti-retroviral therapy
CDR (P+): Copy difference in reference active arm
CDR (P-): Copy difference in reference placebo arm
CR (P+): Copy reference active arm
CR (P-): Copy reference placebo arm
J2R (P+): Jump to reference active arm
J2R (P-): Jump to reference placebo arm
LMCF: Last mean carried forward
MAR: Missing at random
MCAR: Missing completely at random
MI: Multiple imputation under MAR
NMAR: Not missing at random
PM-MI: Pattern-mixture model with multiple imputation
